# Supplementary material for: Transcriptomic Insights into Mechanisms of Early Seed Maturation in the Garden Pea (Pisum sativum L.)
Source: Cells. 2020 Mar 23;9(3):779. doi: 10.3390/cells9030779 (PMC7140803; doi:10.3390/cells9030779)
Supplement: Supplementary file 1 [file cells-09-00779-s001.zip › Supplementary materials/FileS2.zip!/FileS1/Summary Sheets - Modules/D-Cluster/Report.pdf]

D−Cluster

landscape

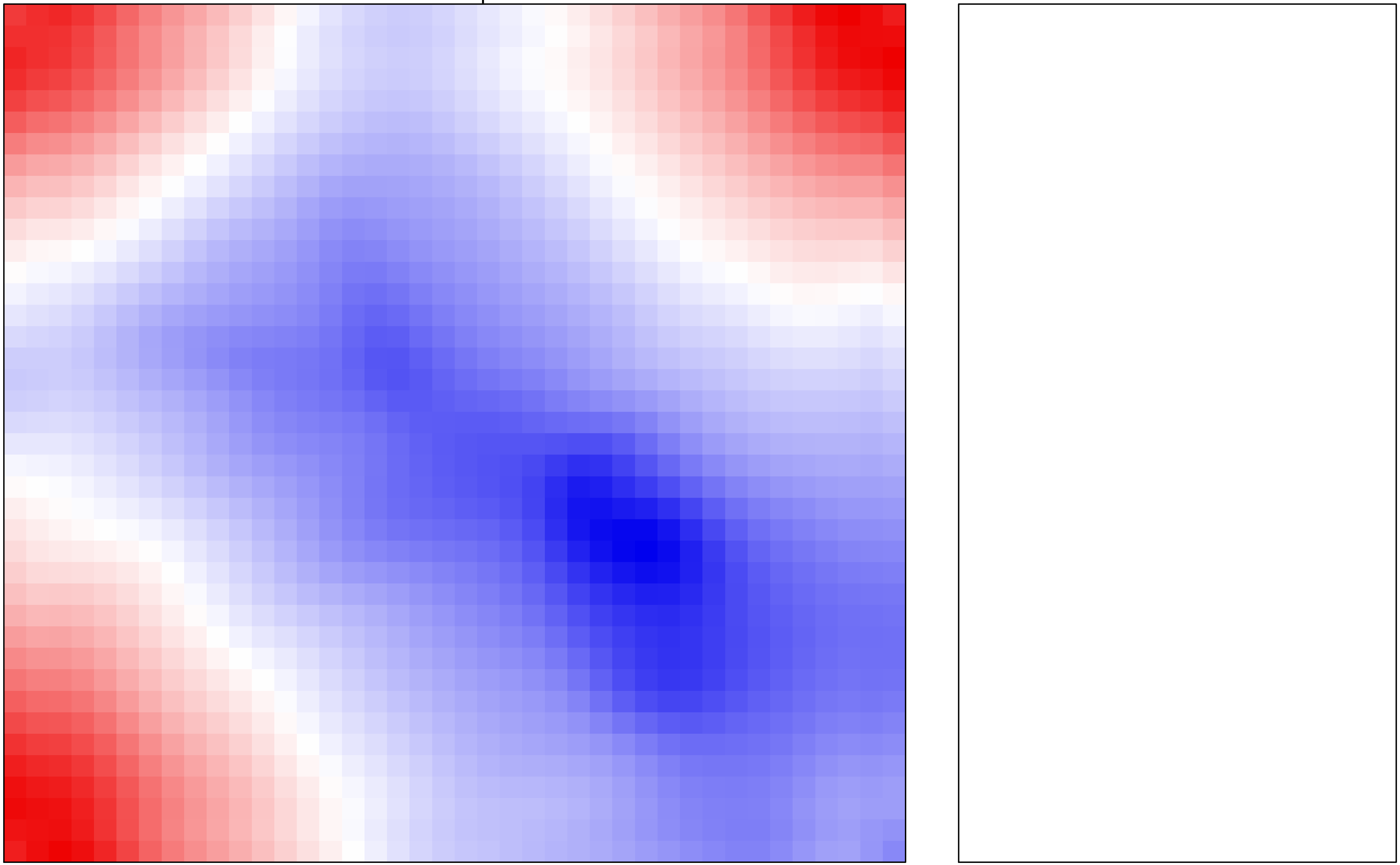

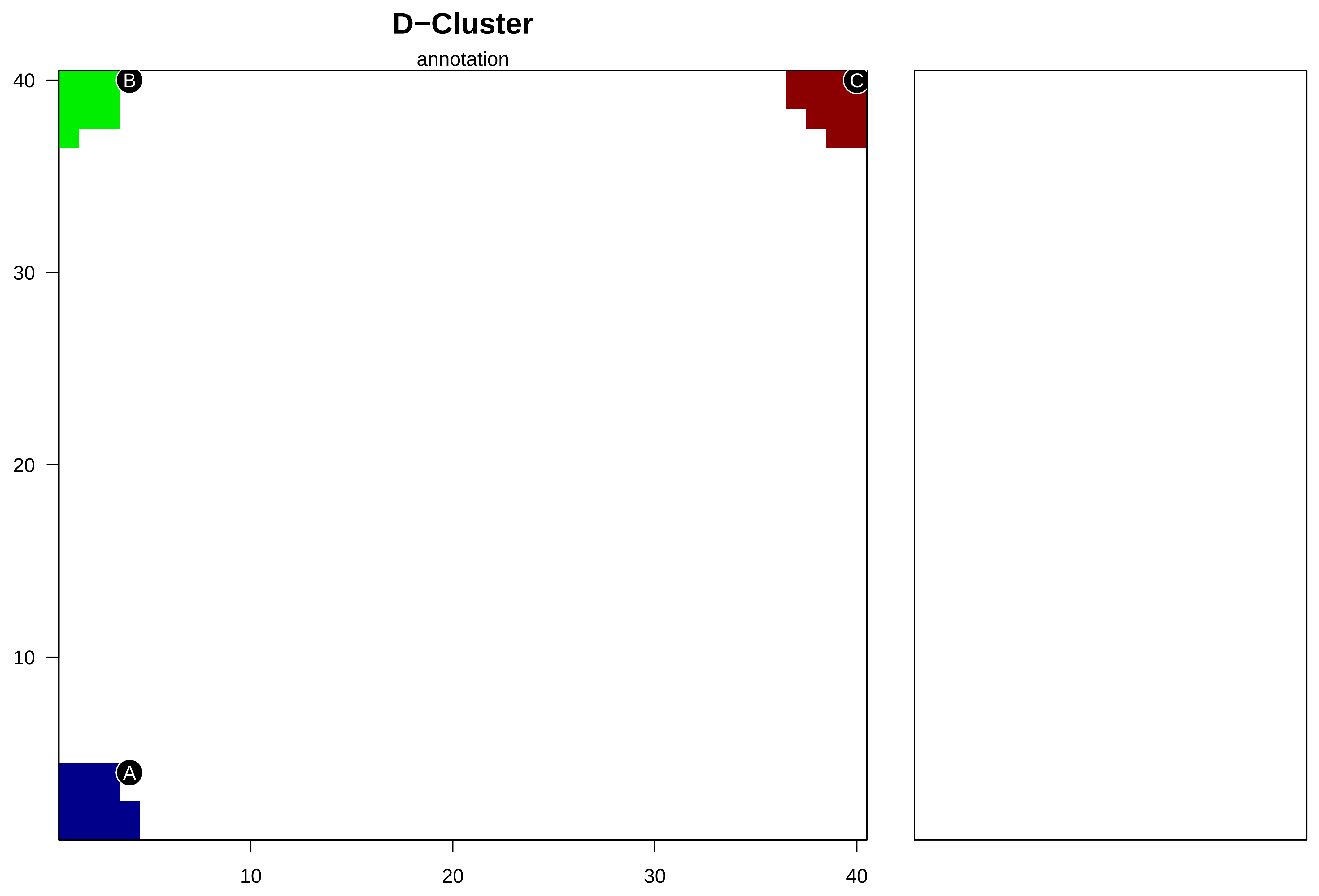
D−Cluster
annotation
40
30
20
10
10
20
30
40
A
B
C

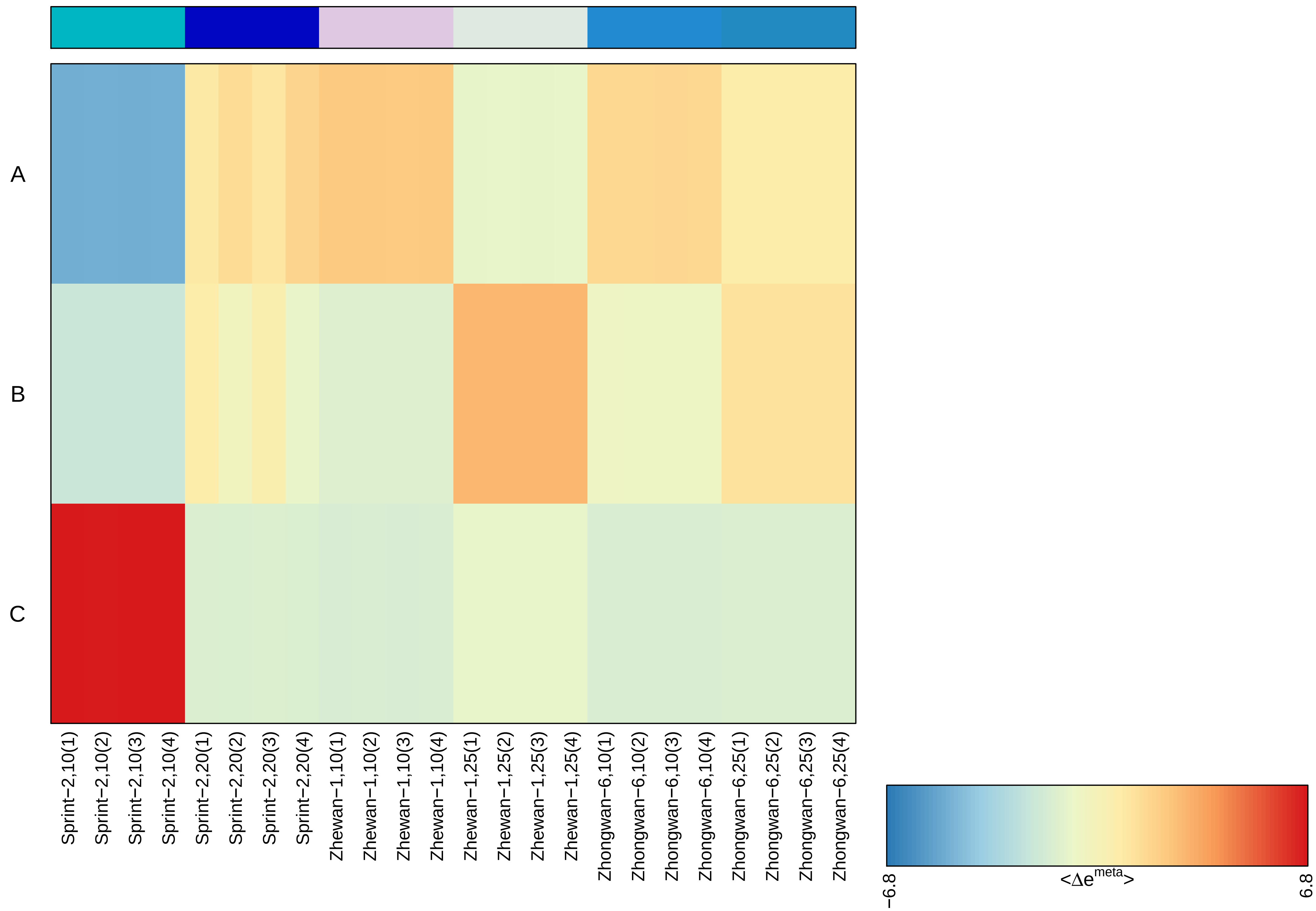
A
B
C
Sprint−2,10(1)
Sprint−2,10(2)
Sprint−2,10(3)
Sprint−2,10(4)
Sprint−2,20(1)
Sprint−2,20(2)
Sprint−2,20(3)
Sprint−2,20(4)
Zhewan−1,10(1)
Zhewan−1,10(2)
Zhewan−1,10(3)
Zhewan−1,10(4)
Zhewan−1,25(1)
Zhewan−1,25(2)
Zhewan−1,25(3)
Zhewan−1,25(4)
Zhongwan−6,10(1)
Zhongwan−6,10(2)
Zhongwan−6,10(3)
Zhongwan−6,10(4)
Zhongwan−6,25(1)
Zhongwan−6,25(2)
Zhongwan−6,25(3)
Zhongwan−6,25(4)
−6.8
<Δe^meta>
6.8

D−Cluster

Spot Summary: A

metagenes = 14
genes = 73

<r> metagenes = 0.99
<r> genes = 0.64
beta: r2= 15.73 / log p= −Inf

samples with spot = 0 (0 %)

Overview Map

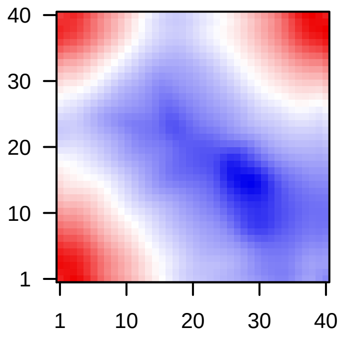

Spot

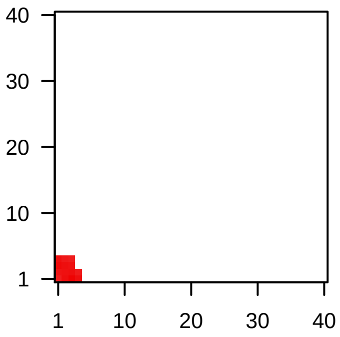

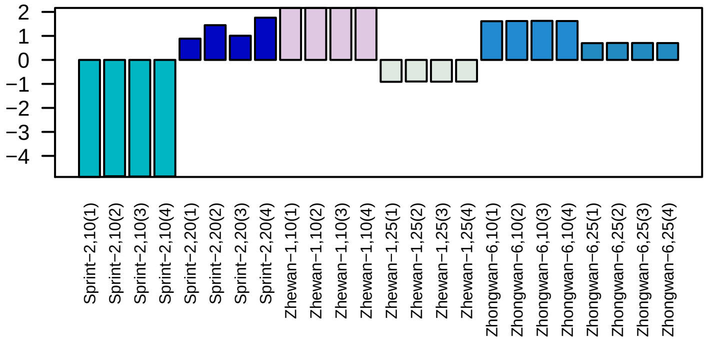

Spot Genelist

Rank	ID	max e	min e	r	Symbol	Description
1	TRINITY_DN2	23.2	−19.57	0.8		
2	TRINITY_DN1	21.33	−106.92	0.91		
3	TRINITY_DN7	20.26	−13.18	0.8		
4	TRINITY_DN4	19.94	−13.52	0.57		
5	TRINITY_DN3	16.43	−9.68	0.65		
6	TRINITY_DN1	13.69	−6.37	0.68		
7	TRINITY_DN7	13.41	−12.69	0.9		
8	TRINITY_DN3	13.38	−29.37	0.89		
9	TRINITY_DN2	11.58	−8.49	0.76		
10	TRINITY_DN3	10.97	−5.32	0.58		
11	TRINITY_DN3	9.96	−9.05	0.55		
12	TRINITY_DN7	7.7	−5.96	0.72		
13	TRINITY_DN3	5.8	−7.86	0.75		
14	TRINITY_DN3	5.67	−6.93	0.78		
15	TRINITY_DN3	5.25	−4.21	0.67		
16	TRINITY_DN3	4.83	−3.23	0.71		
17	TRINITY_DN3	4.13	−3.93	0.78		
18	TRINITY_DN1	4.04	−4.03	0.76		
19	TRINITY_DN6	3.63	−3.54	0.88		
20	TRINITY_DN1	3.44	−2.28	0.78		

D−Cluster

Spot Summary: B

metagenes = 11
genes = 45

<r> metagenes = 0.99
<r> genes = 0.69
beta: r2= 5.21 / log p= −Inf

samples with spot = 4 (16.7 %)
Zhewan−1(25) : 4 (100 %)

Spot Genelist

Rank	ID	max e	min e	r	Symbol	Description
1	TRINITY_DN2	19.02	−23.73	0.93		
2	TRINITY_DN6	13.45	−12.66	0.96		
3	TRINITY_DN2	10.34	−9.73	0.92		
4	TRINITY_DN4	10.09	−9.97	0.93		
5	TRINITY_DN4	9.16	−4.51	0.89		
6	TRINITY_DN2	8.02	−4.57	0.99		
7	TRINITY_DN2	7.51	−8.63	0.91		
8	TRINITY_DN4	6.31	−3.15	0.95		
9	comp39373_c	6.03	−3.43	0.99		
10	TRINITY_DN2	3.51	−1.01	0.89		
11	TRINITY_DN3	2.68	−3.01	0.61		
12	TRINITY_DN1	2.46	−0.77	0.79		
13	TRINITY_DN3	2.45	−1.45	0.99		
14	TRINITY_DN1	2.42	−1.79	0.86		
15	TRINITY_DN1	2.2	−0.88	0.73		
16	TRINITY_DN1	1.98	−1.06	0.51		
17	TRINITY_DN3	1.73	−0.78	0.92		
18	TRINITY_DN2	1.69	−1.29	0.89		
19	TRINITY_DN7	1.61	−1.45	0.71		
20	TRINITY_DN5	1.61	−0.68	0.76		

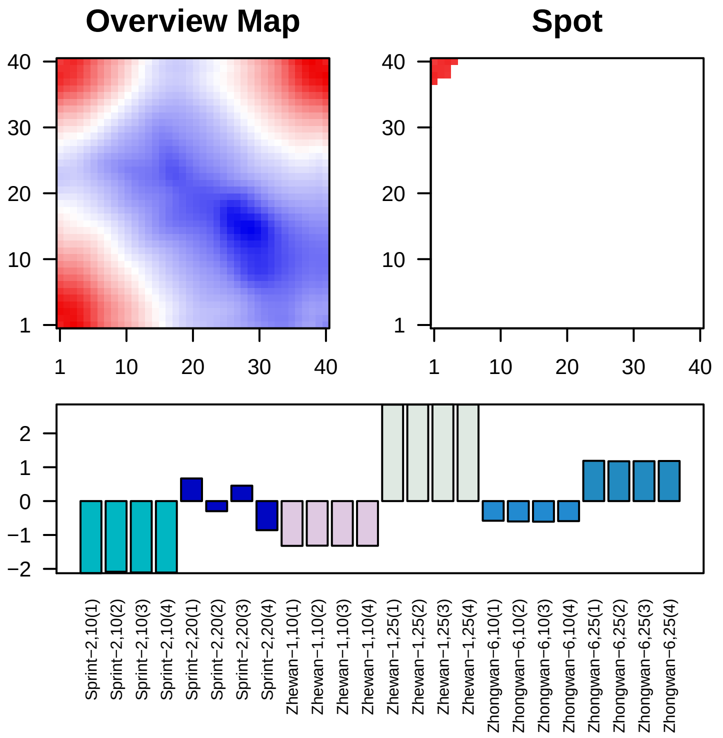

D−Cluster

Spot Summary: C

metagenes = 13
genes = 60

<r> metagenes = 1
<r> genes = 0.87
beta: r2= 24.32 / log p= −Inf

samples with spot = 4 (16.7 %)
Sprint−2(10) : 4 (100 %)

Overview Map

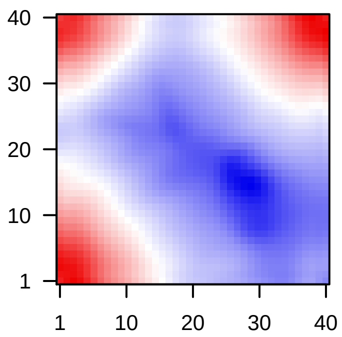

Spot

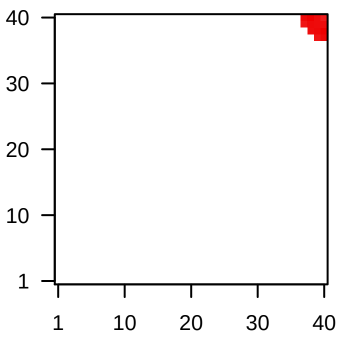

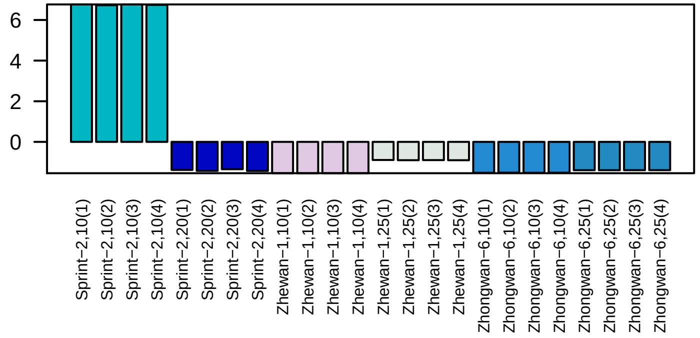

Spot Genelist

Rank	ID	max e	min e	r	Symbol	Description
1	TRINITY_DN7	117.83	−10.22	0.78		
2	TRINITY_DN2	109.03	−18.72	0.93		
3	TRINITY_DN1	36.31	−5.78	0.97		
4	TRINITY_DN8	27.57	−5.74	0.99		
5	TRINITY_DN4	22.28	−3.73	0.99		
6	TRINITY_DN3	14.63	−3.64	1		
7	TRINITY_DN3	13.41	−2.83	0.98		
8	TRINITY_DN8	11.22	−3.35	0.98		
9	TRINITY_DN3	11.09	−2.42	0.98		
10	TRINITY_DN2	10.84	−1.3	0.81		
11	TRINITY_DN5	10.56	−2.04	0.96		
12	TRINITY_DN1	10.49	−2.06	1		
13	TRINITY_DN3	9.42	−1.6	0.98		
14	TRINITY_DN5	9.26	−1.71	0.71		
15	TRINITY_DN1	8.44	−1.61	0.96		
16	TRINITY_DN1	7.68	−1.68	0.96		
17	TRINITY_DN4	7.37	−1.78	0.96		
18	TRINITY_DN1	7.34	−2.04	0.67		
19	TRINITY_DN7	6.84	−1.21	0.99		
20	TRINITY_DN1	6.54	−1.49	0.99		
